# Synthesis and Characterization of Organic Peroxides from Monoterpene-Derived Criegee Intermediates in Secondary Organic Aerosol

**DOI:** 10.1021/acs.est.3c07048

**Published:** 2024-02-07

**Authors:** Kangwei Li, Julian Resch, Markus Kalberer

**Affiliations:** Department of Environmental Sciences, University of Basel, Basel 4056, Switzerland

**Keywords:** organic peroxide, monoterpene-derived SOA, LC-HRMS, kinetic, hydrolysis

## Abstract

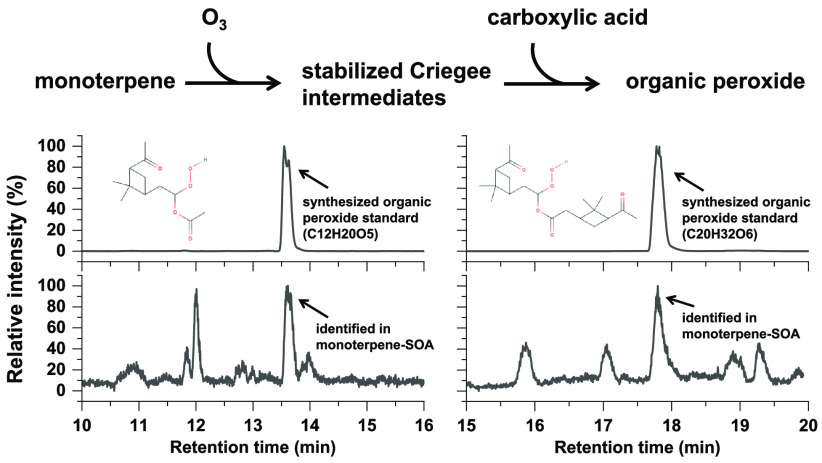

Ozonolysis of alkenes is known to produce reactive intermediates—stabilized Criegee intermediates (SCIs), and their subsequent bimolecular reactions with various carboxylic acids can form α-acyloxyalkyl hydroperoxides (AAHPs), which is considered a major class of organic peroxides in secondary organic aerosol (SOA). Despite their atmospheric and health importance, the molecular-level identification of organic peroxides in atmospheric aerosols is highly challenging, preventing further assessment of their environmental fate. Here, we synthesize 20 atmospherically relevant AAHPs through liquid-phase ozonolysis, in which two types of monoterpene-derived SCIs from either α-pinene or 3-carene are scavenged by 10 different carboxylic acids to form AAHPs with diverse structures. These AAHPs are identified individually by liquid chromatography coupled with high-resolution mass spectrometry. AAHPs were previously thought to decompose quickly in an aqueous environment such as cloud droplets, but we demonstrate here that AAHPs hydrolysis rates are highly compound-dependent with rate constants differing by 2 orders of magnitude. In contrast, the aqueous-phase formation rate constants between SCI and various carboxylic acids vary only within a factor of 2–3. Finally, we identified two of the 20 synthesized AAHPs in α-pinene SOA and two in 3-carene SOA, contributing ∼0.3% to the total SOA mass. Our results improve the current molecular-level understanding of organic peroxides and are useful for a more accurate assessment of their environmental fate and health impact.

## Abstract 2

Molecular-level understanding of particle-phase organic peroxides is limited. This study reports the synthesis, identification, quantification, formation, and hydrolysis kinetics of atmospherically relevant organic peroxides, with implications on their environmental fate.

## Introduction

It is well recognized that secondary organic aerosols (SOA) represent a major fraction of tropospheric fine particles that contribute to serious air pollution, damage human health, and affect Earth’s climate.^1−3^ SOA typically originate from complex atmospheric (photo)chemical oxidation processes of volatile organic compounds, which are emitted from natural and manmade sources.^4−6^ It has been suggested that organic peroxides (ROOR, where R denotes H or an organic group), a major class of SOA components, can significantly contribute to aerosol toxicity and related health effects.^7,8^ This is mainly due to their oxidizing properties, and thus peroxides are a compound class contributing to the so-called reactive oxygen species (ROS), which also includes oxygen-centered inorganic and organic radicals.^9,10^ Despite their atmospheric and health importance, the analytical identification and quantification of compound-specific organic peroxides in atmospheric aerosols is highly challenging, due to their labile properties, complex composition, and limited availability of chemical standards.^11^

The ozonolysis of alkenes generates a type of reactive intermediates, stabilized Criegee intermediates (SCIs), which can further undergo various bimolecular reactions with water, alcohols, aldehydes, and carboxylic acids to form different organic peroxides including α-substituted hydroperoxides and secondary ozonides.^7^ Among those different reactions, α-acyloxyalkyl hydroperoxides (AAHPs) formed through SCIs with carboxylic acids are considered a major class of organic peroxides.^12^ The gas-phase bimolecular reaction of SCIs with carboxylic acids, leading to the AAHP formation, is assumed to be extremely fast and close to the collision limit.^13,14^ While one recent theoretical study^12^ provides some estimated kinetics of larger SCIs (including monoterpene SCIs) with different carboxylic acids in the gas phase, the available direct kinetic measurements for such bimolecular reactions are still limited to C_1_–C_4_ SCIs with C_1_–C_2_ carboxylic acids, with gas-phase rate constants in the range of 1.1 × 10^–10^ to 5 × 10^–10^ cm^3^ molecule^–1^ s^–1^ according to the latest IUPAC evaluation report.^15^ Such limited kinetic information makes it difficult to further constrain important formation pathways of AAHPs given the wide range of structures of both SCIs and carboxylic acids present in the atmosphere (resulting in numerous possible combinations of such bimolecular reactions), especially for large SCIs such as C_10_ SCIs derived from monoterpenes. On the other hand, fast hydrolysis has recently been observed for a few AAHPs and hydrolysis is proposed as their major removal pathway.^16^ This motivated us to perform a more comprehensive evaluation of AAHP formation and hydrolysis kinetics. In summary, the limited availability of AAHP standards and the large uncertainties in our understanding of their formation and loss processes are significant knowledge gaps, which limit our understanding of atmospheric processes of organic peroxides and prevent further assessment of their health effects.

In this study, we synthesize 20 atmospherically relevant organic peroxides, AAHPs, through liquid-phase ozonolysis (using acetonitrile as solvent) of two monoterpenes, α-pinene and 3-carene, in the presence of a mixture of 10 structurally diverse carboxylic acids. The synthesized AAHP standards are identified with liquid chromatography coupled to high-resolution mass spectrometry (LC-HRMS) and their formation and hydrolysis kinetics are investigated. In addition, 4 out of 20 synthesized AAHPs are unambiguously identified in monoterpene-SOA samples generated in laboratory flowtube experiments.

## Materials and Methods

### Chemicals

All chemicals were used as purchased, and abbreviations used herein are given in parentheses. The 10 selected carboxylic acids for AAHPs synthesis include acetic acid (Optima LC/MS grade, Fisher Scientific), pyruvic acid (98%, Sigma-Aldrich), terebic acid (98%, Sigma-Aldrich), adipic acid (99%, Sigma-Aldrich), 3-methyl-1,2,3-butanetricarboxylic acid (MBTCA, 98%, Toronto Research Chemicals), 4-hydroxybenzoic acid (4HA, 99%, Sigma-Aldrich), terephthalic acid (TA, 98%, Sigma-Aldrich), cis-pinic acid (pinic, 99%, Toronto Research Chemicals), cis-pinonic acid (pinonic, 98%, Sigma-Aldrich), and 3-caronic acid (caronic, 95%, Interbioscreen ltd). Additional chemicals used in this study include 2-hydroxyterephthalic acid (TAOH, 97%, Sigma-Aldrich), α-pinene (98%, Sigma-Aldrich), 3-carene (95%, Sigma-Aldrich), acetonitrile (ACN, Optima LC/MS grade, Fisher Scientific), formic acid (Optima LC/MS grade, Fisher Scientific), methanol (Optima LC/MS grade, Fisher Scientific), and water (Optima LC/MS grade, Fisher Scientific).

### LC-HRMS

All samples (injection volume was typically 1 μL) were analyzed by LC-HRMS, consisting of an ultraperformance liquid chromatography unit (ACQUITY UPLC I-Class, Waters) coupled with a high-resolution mass spectrometer (HRMS, Orbitrap Q Exactive Plus, Thermo Scientific). Analytes were separated using a Waters HSS T3 UPLC column (100 mm × 2.1 mm, 1.8 μm) at a temperature of 40 °C and a flow rate of 300 μL min^–1^. The mobile phases include (A1) 10 mM acetic acid in water, (A2) 0.1% formic acid in water, and (B) methanol. Mobile phases A1 and B were used for negative mode analyses, while mobile phases A2 and B were used for positive mode analyses. The two mobile phases A (A1 and A2) were not mixed, and they were switched between negative and positive modes with the same gradient elution program (see below) to maximize the corresponding ionization efficiency. The gradient elution procedure used in this study is similar to that described in our previous study.^17^ Briefly, gradient elution was performed by the A/B mixture at a total flow rate of 0.3 mL min^–1^ for 30 min: 0–1 min at 99.9% A, 1–26 min with a linear gradient to 99.9% B, 26–28 min held at 99.9% B, 28–30 min back to initial condition at 99.9% A for column re-equilibration. The parameters for mass spectrometry were the same for electrospray ionization (ESI) negative and positive modes, which were detailed as follows: spray voltage of 3.4 kV, sheath gas flow of 60, auxiliary gas flow of 15, sweep gas flow of 1, capillary temperature of 320 °C, and auxiliary gas heater temperature of 150 °C. The scan parameters were set to full MS mode, scan range from *m*/*z* 85 to 1000 with a resolution of 70,000 at *m*/*z* = 200, automated gain control (AGC) target of 3E6, and a maximum injection time of 25 ms. The mass spectrometer was calibrated daily for positive and negative modes using Thermo Scientific Pierce Ion Calibration Solution (Fisher Scientific). In addition, an HPLC Gradient System Diagnostics Mix (Sigma-Aldrich) containing five compounds was injected daily to monitor the stability of the signal intensity and retention time (RT), and the measurement uncertainties of these were overall below 4% (Table S1). The LC-HRMS raw data files were converted to mzML format using the ProteoWizard (MSConvert, version 3) software^18^ and were subsequently analyzed in RStudio (R 4.2.1, Boston, MA) using the peakPantheR package^19^ for targeted peak extraction. The extracted ion chromatograms (EICs) for selected ions were exported using Xcalibur 2.2 software (Thermo Scientific) with a mass tolerance of 10 ppm.

### AAHPs Synthesis through Liquid-Phase Ozonolysis

**Scheme 1:**
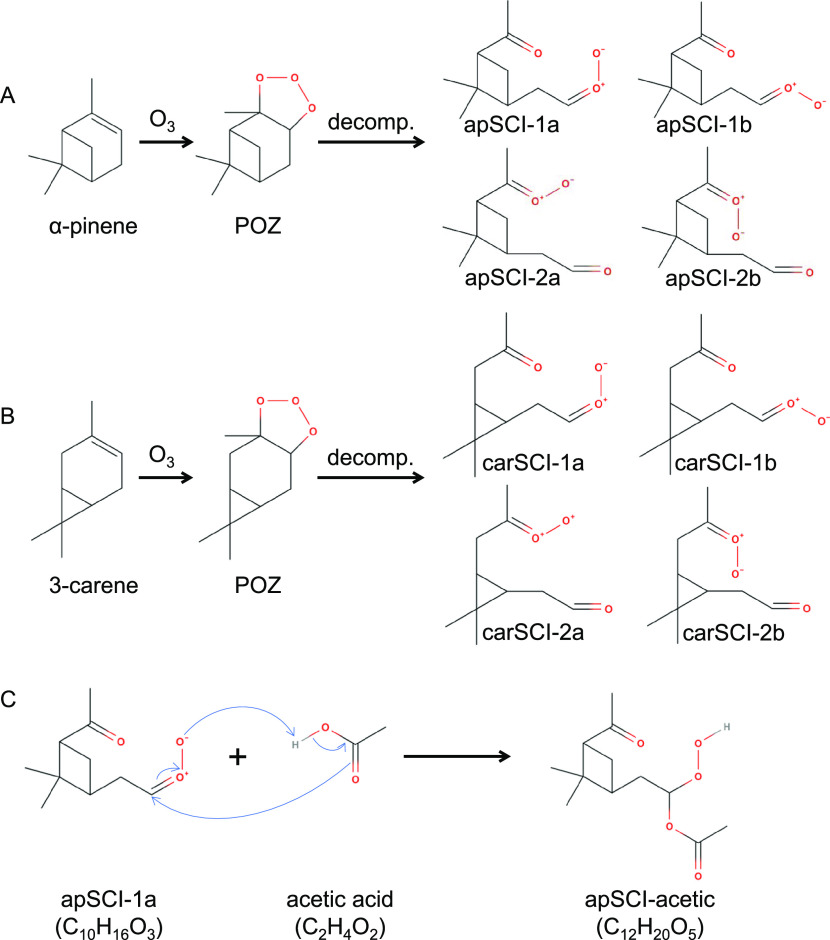
Reactions Illustrating Ozonolysis (A) α-Pinene and (B) 3-carene to form their corresponding primary ozonide (POZ), followed by rapid decomposition of POZ to produce type 1 and type 2 SCIs, with each type of SCI also containing *syn*- and *anti*-SCI. (C) Reaction between apSCI-1a and acetic acid as an example of AAHP formation. Note that the other three structures of apSCI-acetic are not shown, and each AAHP standard synthesized in this study should have four structures.

We used an impinger to synthesize a number of AAHPs via liquid-phase ozonolysis following existing methods^20,21^ and the setup displayed in Figure S1A. Specifically, individual carboxylic acids or a mixture of 10 carboxylic acids (see Table S2 for experiment summary) were added into acetonitrile solutions containing either α-pinene or 3-carene. Then, high concentrations of O_3_ (∼500 ppm), which was generated by irradiating a flow of clean air (100 mL min^–1^) with a UV lamp (Pen-Ray photochemical quartz lamp), were bubbled through these solutions. O_3_ reacted in solution with α-pinene or 3-carene resulting in the formation of SCIs, which are rapidly scavenged by the added carboxylic acid to form the corresponding AAHPs, according to generally accepted reaction mechanisms as displayed in Scheme 1.^12,15,22^ For simplification, apSCI and carSCI, as well as apAAHP and carAAHP, refer to SCIs and AAHPs from α-pinene and 3-carene, respectively. The individual name of the synthesized AAHPs in this study follows the rule by combining a specific SCI and carboxylic acid, i.e., apSCI-acetic, carSCI-pinic, etc. We choose two structurally similar monoterpenes (α-pinene and 3-carene) and expect comparable properties between the two types of AAHPs (i.e., apAAHPs vs carAAHPs).

Some bubbling experiments (expt 15–18 in Table S2) were also designed for kinetic studies where a mixture of 10 carboxylic acids was added to the monoterpene solutions. At each time interval, 75 μL of solution was taken from the impinger and stored in amber vials with inserts (200 μL, Waters) for LC-HRMS injection. Such kinetic experiments lasted for 15 min and provided decreasing concentration profiles of individual carboxylic acids due to their bimolecular reactions with SCIs. Note that we did not add ozone scavenger after the reaction, thus there might be leftover ozone, but this should have negligible influence on our main results (see details in Text S1). To avoid the hydrolysis of AAHPs during the synthesizing process, all bubbling experiments were performed in acetonitrile as solvent. In addition, the high acetonitrile in the bubbling experiments assures that >99% of OH radicals (formed during the decomposition of Criegee intermediates) are scavenged by acetonitrile and do not react further with other organic compounds in the impinger, as described in detail in Supporting Information Text S2. Measurement for hydrolysis purpose was made by mixing the synthesized AAHP mixture solution with water (v/v = 1/9), followed by seven sequential LC-HRMS injections with an injection volume of 5 μL. This allows the determination of hydrolysis rates of AAHPs. The sequential injections mentioned above were done for two different temperatures, where the temperature in the autosampler was set to either 20 or 8 °C.

### SOA Generation in Flowtube Experiments

We performed flowtube experiments by mixing either α-pinene or 3-carene vapor with O_3_ in the dark to generate corresponding SOA particles, which were collected onto 47 mm PTFE membrane filters (0.2 μm pore size, Whatman) with a known mass loading. Figure S1B shows the flowtube setup, which includes a recently developed instrument—organic coating unit^23^—for controlled and constant generation of monoterpene vapor before mixing with O_3_. The initial flowtube conditions are summarized in Table S8. After collection, one-fourth of the filter was immediately extracted in acetonitrile. The extracts were then concentrated to complete dryness (Eppendorf Concentrator plus, Fisher Scientific) and reconstituted with 100 μL of acetonitrile before being injected into LC-HRMS. To check for possible artifacts of drying down samples, we also tested the SOA filter extraction in 1.5 mL of ACN without drying, which has a solvent volume difference by a factor of 15 compared to the other extraction method. As shown in Table S3, the comparison between the two types of extractions generally shows a factor of 10–15 difference.

### UV–Vis and Fluorescence Spectroscopy

A blank experiment was performed by bubbling ozone through an acetonitrile solution and measuring the ozone concentration with UV–vis spectroscopy (LAMBDA 365, PerkinElmer) to assess the solubility of ozone in acetonitrile. Figure S2 displays the absorption spectra of O_3_ with a known peak at 260 nm,^24^ which suggests that the gaseous O_3_ is dissolved in acetonitrile solution to trigger the liquid-phase ozonolysis. Commercial fluorescence spectroscopy (Fluorolog-3, Horiba Jobin Yvon) was used to measure TAOH—a fluorescent product from the reaction of TA with OH radical (see Text S2 for details).

## Results and Discussion

### Synthesis and Identification of AAHPs

As described above, 20 atmospherically relevant AAHP standards were synthesized by reacting 10 carboxylic acids with SCIs from either α-pinene or 3-carene. Table S4 summarizes the carboxylic acids ranging from C_2_ to C_10_ with diverse chemical structures. They are selected not only because of their high atmospheric abundance but also to cover both anthropogenic and biogenic sources, as well as a variability of functional groups in the class of carboxylic acids. For example, acetic acid and pyruvic acid are ubiquitous in both gas phase and aqueous phase of the troposphere, and they are also abundant degradation products or organic compounds from both anthropogenic and biogenic sources.^25^ Adipic acid, terephthalic acid, and 4-hydroxybenzoic acid are mainly emitted from anthropogenic sources such as biomass burning and combustion,^26,27^ while terebic acid, MBTCA, cis-pinic acid, cis-pinonic acid, and 3-caronic acid are known atmospheric oxidation products from biogenic monoterpenes.^28,29^

**Figure 1:**
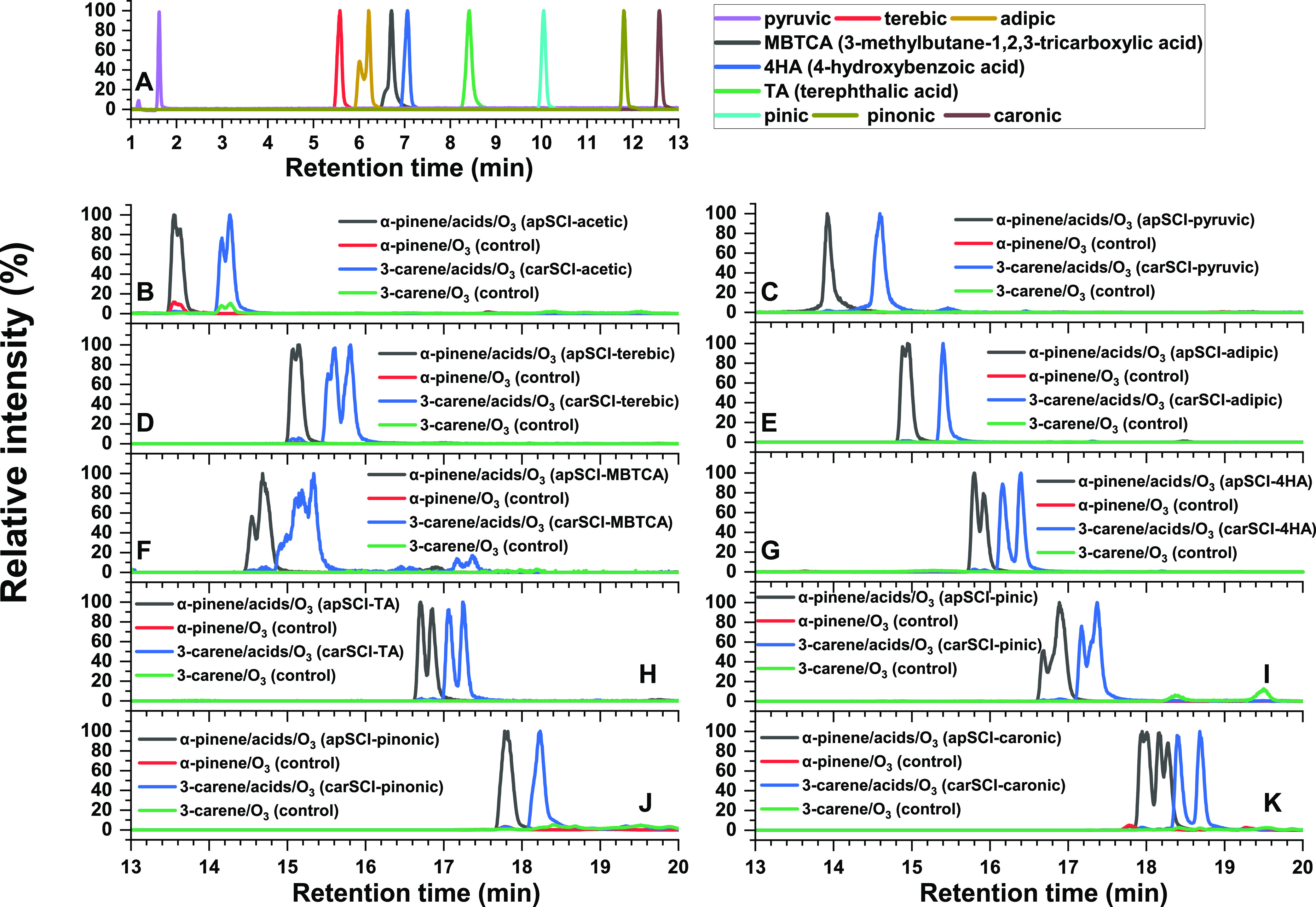
Extracted ion chromatograms (EICs) for (A) carboxylic acid standards and (B–K) synthesized AAHP standards. Their retention time (RT) and other details are summarized in Tables S4 and S5.

Figure 1A shows the EICs of carboxylic acid standards detected as [M – H]^−^ in negative ionization mode. Acetic acid is not detected with our method because mobile phase solvent A1 contains 10 mM acetic. Given the ultrahigh resolving power of the mass spectrometer used in this study, the 20 synthesized AAHP standards were unambiguously identified individually in positive mode (detected as [M + Na]^+^) and clearly separated with the chromatographic method used here, especially when comparing with the control experiments without adding carboxylic acid standards, as displayed in Figure 1B–K. In addition, we employed an iodometry-assisted LC-HRMS method proposed by Zhao et al.^21^ to the synthesized AAHP standards. As expected, we observed the disappearance of these chromatographic peaks of AAHPs in iodometry-treated samples (see details in Text S3 and Figure S3), which again confirms the unambiguous identification of AAHPs.

Tables S4 and S5 summarize the retention time (RT) for all of the detected peaks of carboxylic acids and AAHPs in Figure 1. In addition to the [M + Na]^+^ adduct, these AAHPs were also detected as K^+^ and NH_4_^+^ adducts in positive mode, while the ionization efficiency follows the order Na^+^ > K^+^ > NH_4_^+^. Some AAHPs were also detected as [M – H]^−^ in negative mode when they contained a carboxylic acid or alcohol functional group. For the LC condition used here, these carboxylic acids elute between 0 and 13 min, while AAHPs elute between 13 and 20 min because of their larger molecular weight. Most of these AAHPs showed two or multiple peaks, suggesting that several isomers were separated. These isomers arise from stereoisomers of SCI, as ozonolysis of either α-pinene or 3-carene results in four stereoisomers of SCI, i.e., the type 1 and type 2 isomers as displayed in Scheme 1. Since α-pinene and 3-carene are two structurally similar monoterpenes, it is expected that the corresponding apAAHPs and carAAHPs have comparable properties as seen, for example, by their usually similar chromatographic elution pattern, where apAAHPs always elute 0.4–0.7 min earlier than carAAHPs. Interestingly, AAHPs generally show a similar eluting sequence as their corresponding carboxylic acids, with reasonably good correlation, as shown in Figure S4. This suggests that these AAHPs maintain a similar order of polarity or solubility in the mobile phases (water and methanol) as their corresponding carboxylic acids.

### Relative Formation Kinetics and Quantification of Individual AAHP

As reactive intermediates, the steady-state concentration of SCIs is usually low, and it is expected that various carboxylic acids competitively participate in bimolecular reactions with SCIs that lead to the formation of AAHPs. Therefore, it is crucial to determine the rate constants between SCIs and various carboxylic acids, which can be achieved based on relative rate method.^30^ Since absolute liquid-phase rate constants for C_10_ SCIs with any carboxylic acid are not available, only a relative comparison of the formation rate constants between C_10_ SCIs and the various carboxylic acids is given here, which can be obtained by measuring the decay profile of individual carboxylic acids during the liquid-phase ozonolysis. This requires that these carboxylic acids do not react with other components in the impinger.

To rule out potential side reactions of carboxylic acids, we carefully define the initial conditions for kinetic experiments and examine the feasibility by considering (i) total SCI yield; (ii) influence of dissolved O_3_; and (iii) influence of OH radicals from the decomposition of Criegee intermediates. As shown in Table S2 (expts 15 and 16), the initial α-pinene or 3-carene concentration for kinetic experiments is 1 mM, while the initial total concentration of the 10 carboxylic acids is ∼0.2 mM (∼0.02 mM of each carboxylic acid). SCIs are formed with a yield of ∼0.19,^22^ and therefore the total amount of SCIs (integrated over the entire reaction time) is ∼0.19 mM, which is equivalent to the total initial concentration of carboxylic acids added to the solution. The dissolved O_3_ might react with carboxylic acids during the O_3_ bubbling process, leading to an additional loss process of the carboxylic acid. However, reaction rate constants between ozone and α-pinene/3-carene or carboxylic acids in acetonitrile are not available in the literature, and even highly sparse in water.^31^ To rule out this possibility, we performed a control experiment by bubbling ozone through an acetonitrile solution that only contains the same mixture of carboxylic acids in the absence of α-pinene and 3-carene. The results displayed in Figure S6 show that all of these carboxylic acids are overall stable in the presence of ozone under the conditions used here, suggesting that evaporation losses of the carboxylic acids or losses due to reaction with ozone are negligible. Other unwanted side reactions might occur between carboxylic acids and OH radicals, which are known to be produced from the decomposition of Criegee intermediates.^15,32^ Because acetonitrile is used as a solvent, most OH radicals (>99%) produced during ozonolysis should be scavenged by acetonitrile based on reactivity calculations, which is further supported by negligible fluorescence signals for an OH-oxidation product from TA (see Text S2 for details). In summary, we can conclude that the decay profiles of carboxylic acids observed in this study are only a result of their bimolecular reactions with SCIs, while unwanted side reactions of carboxylic acids with the OH and O_3_ radicals are negligible.

**Figure 2:**
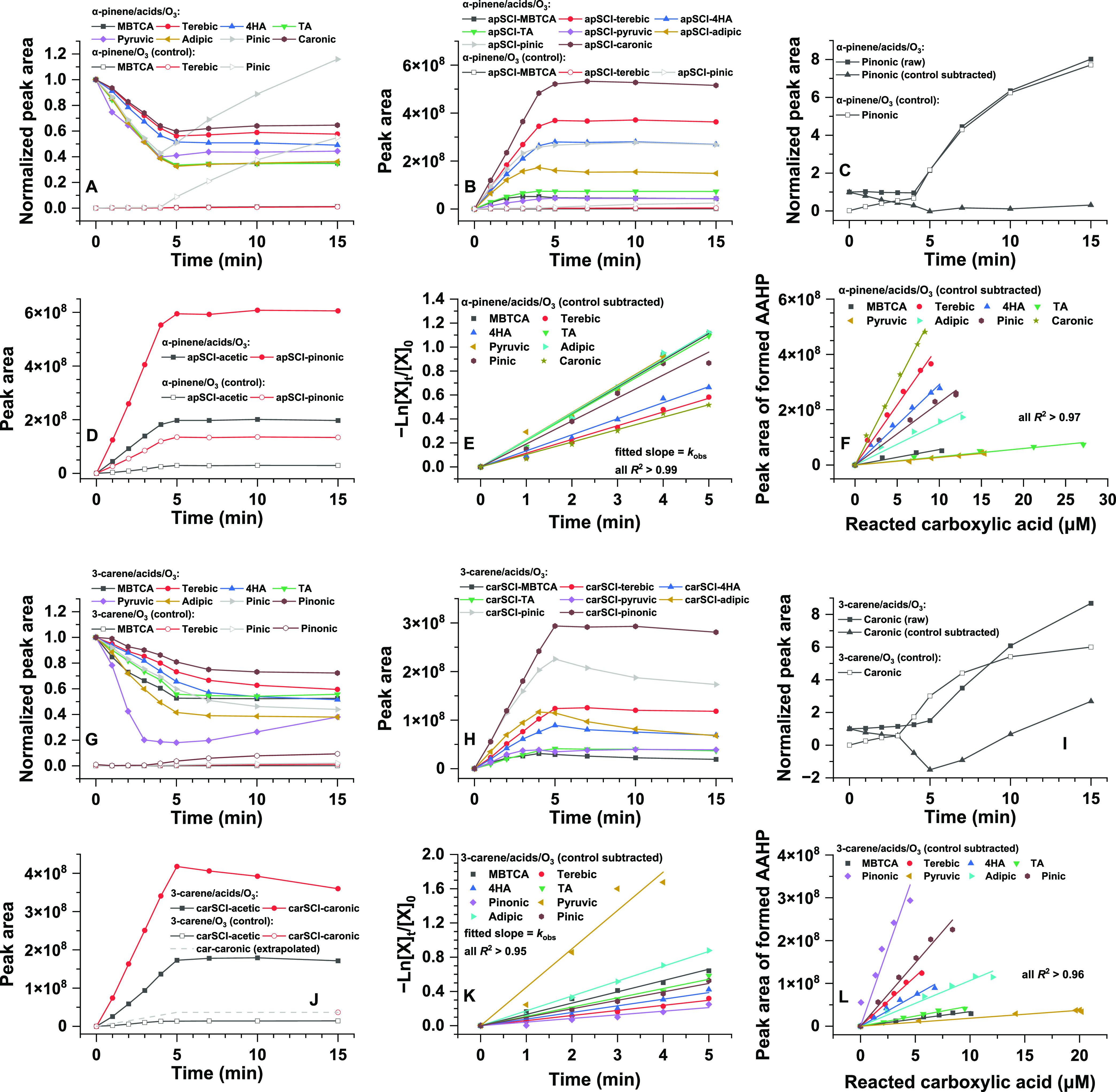
(A–D) Temporal profiles of carboxylic acids and the corresponding AAHPs during liquid ozonolysis of α-pinene in the presence and absence of the 10 carboxylic acids mixture; (E) natural log normalized carboxylic acids derived from (A), where the slopes of the linear fits are the respective rate constants (*k*_obs_) between apSCIs and various carboxylic acids, assuming pseudo-first-order kinetics; (F) correlation between the measured consumption of carboxylic acids and their corresponding apAAHP formation; and (G–L) same as (A–F) but for 3-carene.

As shown in Figure 2A,C, most of the carboxylic acids decay over time to 40–60% of the initial concentrations and then reach a plateau after ca. 5 min, while the formation of all of the apAAHPs also reaches a plateau after ca. 5 min (Figure 2B,D). This suggests that apSCIs are no longer available and that SCI-involved bimolecular reactions are finished within ca. 5 min. Since terebic acid, cis-pinic acid, cis-pinonic acid, and MBTCA are also oxidation products of α-pinene,^28^ it is necessary to determine whether these products are produced over a time frame of 15 min and whether this might affect their observed decay profiles. The control experiment shows that only marginal concentrations of terebic acid and MBTCA are formed within 15 min, while cis-pinic acid is not observed until 4 min (Figure 2A). This suggests that the kinetics for terebic acid, cis-pinic acid, and MBTCA can still be determined after subtraction of control experiments. cis-Pinonic acid is an exception because it is formed from the very beginning of the reaction with high production yield (Figure 2C); thus, the kinetics for cis-pinonic acid cannot be reliably retrieved due to this significant interference.

Figure 2G–L shows the same temporal profiles for carboxylic acids and carAAHPs from 3-carene experiments, which overall displays a behavior similar to the α-pinene experiments, although some individual profiles remain unexplainable. Similar to the above, 3-caronic acid as a known oxidation product from 3-carene ozonolysis is the exception among these carboxylic acids because of its high production yield from 3-carene. Thus, its kinetics with carSCI cannot be reliably obtained.

Figure 2E,K shows the temporal variation of the different carboxylic acids relative to the starting concentration. Assuming pseudo-first-order kinetics, the slopes of the fits provide a relative comparison of the reaction rate constants between SCIs and the eight acids, which are summarized in Table S6. Normalizing these rate constants illustrates that they differ only within a factor of 2–3, except carSCI-pyruvic as an outlier. Such small differences suggest that there is little chemical selectivity in the reaction between SCIs and various carboxylic acids in the aqueous phase, which may be extrapolated to gas-phase reactions. Chhantyal-Pun et al.^12^ summarize gas-phase rate coefficients between C_1_–C_10_ SCIs (*n* = 14) and C_1_–C_10_ carboxylic acids (*n* = 9), which were estimated using a structure–activity relationship. Except two carboxylic acids in their study, the predicted gas-phase rate constants also show a small variation, within a factor of 1–5 for various SCIs and carboxylic acids.^12^

The kinetic control experiments described above have ruled out other significant chemical sources and sinks for these eight carboxylic acids and show that their decay profiles are only related to the bimolecular reactions with SCIs that lead to AAHP formation. If we assume the AAHPs are stable in acetonitrile (which is demonstrated in the next section), then we can assume a 1:1 ratio between the molar consumption of carboxylic acid and formation of AAHP. This allows us to quantify AAHP concentrations, as the consumption of carboxylic acid can be determined using the calibration curves shown in Figure S5. The good correlations between chromatographic peak areas of AAHPs and their corresponding reacted carboxylic acids from the same ozonolysis reaction time (Figure 2F,L) demonstrate the validity of this approach, where the slopes represent the calibration factors to quantify individual AAHPs (Figure S8). To the best of our knowledge, this is the first time that individual AAHPs are quantified. We note that apAAHPs and carAAHPs formed from the same carboxylic acid show similar calibration factors, which agree with the hypothesis of a comparable property between these two structurally similar AAHPs.

### Hydrolysis of AAHP

Previous studies reported that AAHPs can undergo fast hydrolysis in water, which was proposed as their major removal pathway in the atmosphere.^16^ Here, we determine the decomposition of all synthesized AAHPs in water (water:ACN = 9:1 (v/v)) and in 100% ACN to mimic their stability in an aqueous or organic medium like cloudwater or organic aerosol, respectively. Figure S9 shows that all of the AAHPs are relatively stable in acetonitrile but decay at different rates in water, suggesting the involvement of hydrolysis reactions. Higher temperature results in a faster hydrolysis rate, with a difference of a factor of ∼3 for 20 vs 8 °C. Additional hydrolysis measurements were performed on two selected solutions containing a single synthesized AAHP, which show overall comparable results with those in AAHP mixture solutions (Figure S10), suggesting that any matrix effects on hydrolysis kinetics are not significant. We also tested the stability of AAHPs in methanol (10% ACN + 90% methanol) at 8 °C. As shown in Figure S11, AAHPs are overall less stable in methanol than ACN, and such solvent-dependent AAHP stability is consistent with previous observations.^16^

Table 1 summarizes the hydrolysis rate constants and the e-folding lifetime for all 20 AAHPs. Overall, hydrolysis lifetimes vary strongly from a few minutes to a day. At the same temperature, the lifetime among these AAHPs still varies over 2 orders of magnitude. Zhao et al.^16^ also investigated the hydrolysis of two individual AAHPs (apSCI-pinonic and apSCI-adipic) over a wide range of conditions, which can be adjusted to conditions (i.e., temperature, solution pH, and dilution factor) used in our study. As shown in Table S7, the hydrolysis rates and e-folding lifetimes of these two AAHPs were very similar between the two studies. By synthesizing a wide range of AAHP structures, our results indicate that the hydrolysis properties of AAHPs are strongly compound-dependent, which increases the complexity of predicting their atmospheric removal fate in the aqueous phase. It should be noted that AAHP hydrolysis can also occur in eluent A during LC-HRMS analysis, resulting in an underestimation for some AAHPs that have extremely fast hydrolysis rates. This is an analytical limitation that cannot be fully avoided.

**Table 1:** AAHP Hydrolysis Rate and e-Folding Lifetime^a^

		apSCI-acetic	apSCI-MBTCA	apSCI-terebic	apSCI-4HA	apSCI-TA	apSCI-pinonic	apSCI-pyruvic	apSCI-adipic	apSCI-pinic	apSCI-caronic
20 °C	*k*_90%H_2_O_ (×10^–5^ s^–1^)	151.6 ± 15	3.6 ± 0.5	146 ± 5.7	4 ± 0.1	25 ± 0.1	97.8 ± 2.4	392.1^b^	82.5 ± 3.1	13.1 ± 0.8	87.6 ± 1.7
	lifetime τ (min)	11 ± 1	459 ± 65	11 ± 0.4	414 ± 7	67 ± 0.2	17 ± 0.4	4^b^	20 ± 1	127 ± 8	19 ± 0.4
8 °C	*k*_90%H_2_O_ (×10^–5^ s^–1^)	56.2 ± 2.6	2.3 ± 0.2	42.5 ± 2	1 ± 0.1	5.9 ± 0.1	33.2 ± 0.7	130.7 ± 3.8	27.5 ± 0.4	6.1 ± 0.4	29 ± 0.6
	lifetime τ (min)	30 ± 1	740 ± 73	39 ± 2	1639 ± 134	284 ± 6	50 ± 1	13 ± 0.4	61 ± 1	273 ± 17	57 ± 1

		carSCI-acetic	carSCI-MBTCA	carSCI-terebic	carSCI-4HA	carSCI-TA	carSCI-pinonic	carSCI-pyruvic	carSCI-adipic	carSCI-pinic	carSCI-caronic
20 °C	*k*_90%H_2_O_ (×10^–5^ s^–1^)	128 ± 8.4	5.5 ± 0.6	98.7 ± 6.9	3.9 ± 0.1	22.8 ± 0.1	62.9 ± 5.1	218.3	62.2 ± 3.4	13.4 ± 1	51 ± 4.6
	lifetime τ (min)	13 ± 1	301 ± 32	17 ± 1	429 ± 8	73 ± 0.4	27 ± 2	8	27 ± 1	124 ± 9	33 ± 3
8 °C	*k*_90%H_2_O_ (×10^–5^ s^–1^)	49.3 ± 2.5	2.9 ± 0.2	36.6 ± 1.7	1 ± 0.1	6.3 ± 0.2	29 ± 0.9	118.2 ± 11	25.1 ± 0.6	7.1 ± 0.4	24.1 ± 0.9
	lifetime τ (min)	34 ± 2	569 ± 32	46 ± 2	1697 ± 150	266 ± 6	57 ± 2	14 ± 1	66 ± 2	234 ± 15	69 ± 3

a Measured in 90% water + 10% ACN. The pH of the two types of AAHP solutions (after mixing with 90% water) was uncontrolled and measured as 4.96 (apAAHPs) and 4.58 (carAAHPs), respectively.

b The hydrolysis rate (392.1 × 10^–5^ s^–1^) and lifetime (4 min) of apSCI-pyruvic at 20 °C is not derived directly from measurement due to its hydrolysis being too fast, and it is estimated from the hydrolysis measurement at 8 °C but considering a factor of 3.

We correlated the measured hydrolysis rate with the retention time (as a proxy to polarity) and the molecular weight of each individual AAHP. As shown in Figure S12, the slightly negative correlation suggests that the AAHP hydrolysis rate is likely linked to individual polarity and molecular weight, but the fundamental reason for explaining their diverse behaviors remains unknown. Interestingly, the two types of AAHPs (apAAHP vs carAAHP) generally show a similar hydrolysis rate when the AAHP was formed from the same carboxylic acid (Table 1). This is again consistent with the hypothesis of a comparable property between apAAHP and carAAHP due to their similar structure.

### Identification of AAHPs in Laboratory-Generated Monoterpene-SOA

**Figure 3:**
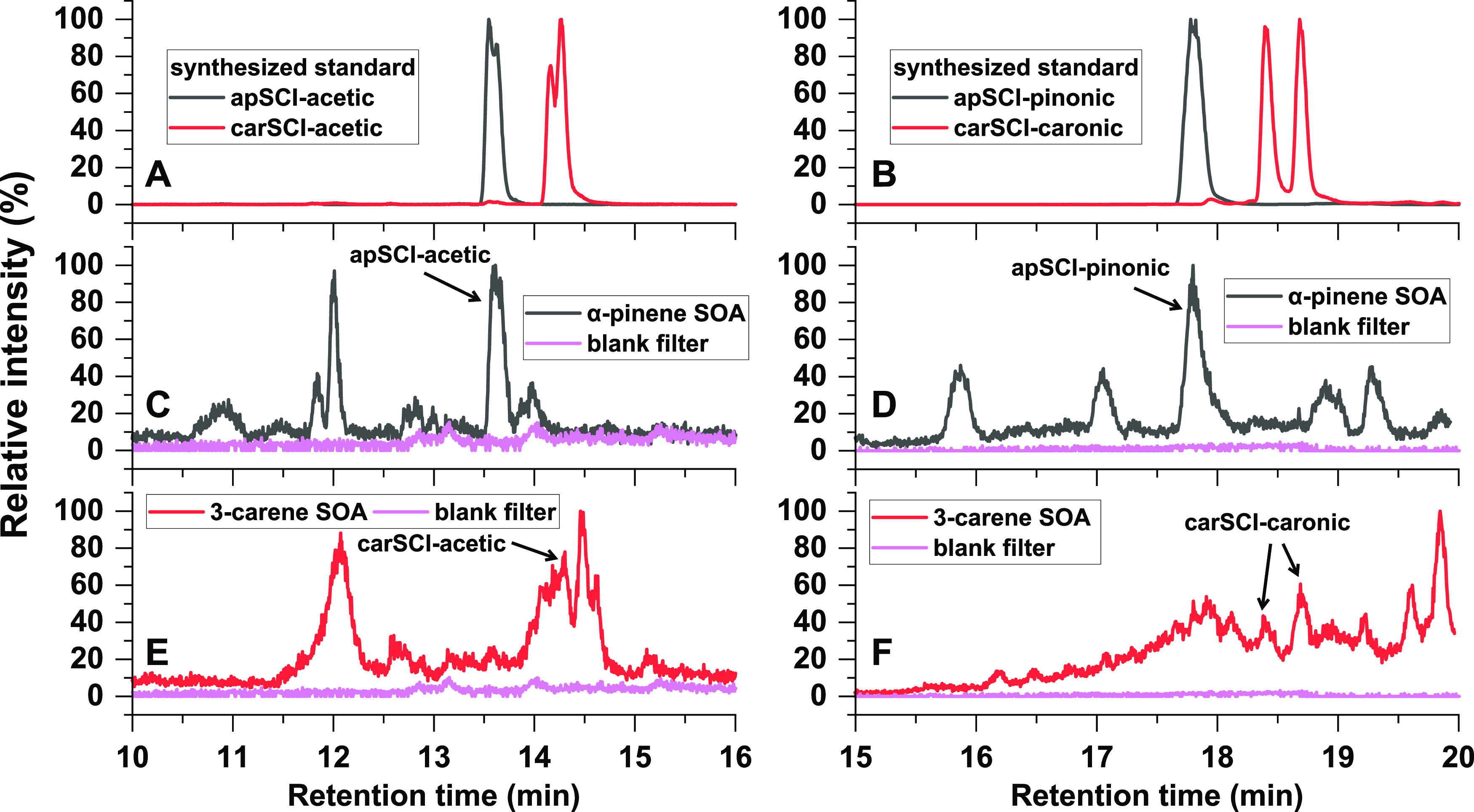
Identification of AAHPs in laboratory-generated α-pinene SOA and 3-carene SOA through comparison with synthesized AAHPs standards. (A, C, E) EIC of *m*/*z* 267.1203 (C_12_H_20_O_5_Na^+^ refers to apSCI-acetic or carSCI-acetic); (B, D, F) EIC of *m*/*z* 391.2091 (C_20_H_32_O_6_Na^+^ refers to apSCI-pinonic or carSCI-caronic).

Acetic acid is a known gas-phase product from α-pinene and 3-carene ozonolysis, with reported high molar yield of 8 and 14% respectively.^33^ Similarly, cis-pinonic acid and 3-caronic acid are structurally similar carboxylic acids that exist in both gas and aerosol phases from α-pinene and 3-carene ozonolysis, and their reported molar yields are 2.2–7.9 and 4.2%, respectively.^34^ Given the high product yields of these carboxylic acids, it is expected that their corresponding AAHPs could be present in α-pinene SOA and 3-carene SOA. To test this possibility, Figure 3 shows a comparison between AAHP standards (same data as shown in Figure 1) and laboratory-generated SOA. Extracted ion chromatograms of α-pinene and 3-carene SOA extracts show several isomers eluting at different RT, but in each SOA type, several chromatographic peaks match reasonably well with the four AAHP standards, i.e., *m*/*z* 267.1203 (C_12_H_20_O_5_Na^+^ refers to apSCI-acetic or carSCI-acetic) or *m*/*z* 391.2091 (C_20_H_32_O_6_Na^+^ refers to apSCI-pinonic or carSCI-caronic). We further compared the MS^2^ pattern of standards and SOA components at identical retention times and found excellent agreement, as displayed in Figure S13. This illustrates that these four AAHPs have been unambiguously identified in our laboratory-generated SOA samples using our synthesized authentic standards.

We also checked other apAAHPs that are formed from terebic acid, cis-pinic acid, and MBTCA with apSCI, as these carboxylic acids are also known oxidation products from α-pinene.^28^ However, these AAHPs could not be identified in α-pinene SOA, suggesting that these carboxylic acids are not major contributors to AAHPs. We notice that a previous study by Zhao et al.^21^ failed to identify organic peroxides (especially AAHPs such as apSCI-acetic and apSCI-pinonic) in their α-pinene SOA sample. This is very likely due to the fast decomposition of these two AAHPs in water as their SOA filter was extracted with water, while we intentionally extracted the filters with acetonitrile to avoid AAHP hydrolysis during our extraction procedure. Note that we used very high concentrations of precursors to generate SOA, which are significantly higher than the α-pinene flowtube experiments performed by Zhao et al.^21^ The different SOA generation conditions between the two studies could be another possible reason for such observed discrepancies.

The calibration factors for these four AAHPs are not available, as detailed in the earlier discussion on AAHP formation kinetics and quantification. Alternatively, we obtained estimated factors by fitting the available calibration factors from 16 AAHPs with their retention time, as illustrated in Figure S8B. This allows us to perform a semiquantitative analysis of the laboratory-generated SOA. As summarized in Table S8, the combined mass contribution of the two identified AAHPs (apSCI-acetic and apSCI-pinonic) to α-pinene SOA was 0.32%, while the other two identified AAHPs (carSCI-acetic and carSCI-caronic) contributed 0.31% to the mass of 3-carene SOA. Conventional iodometry measurements for α-pinene SOA using UV–vis spectroscopy suggest that the mass yield of total peroxides in SOA is around 15–20%, which typically includes both organic hydroperoxides and H_2_O_2_ and are usually not differentiated.^21,35^ Nevertheless, this is clearly a large discrepancy with the estimated mass contribution (i.e., 0.3%) from this study, illustrating the need to expand the molecular-level identification and quantification ability of organic peroxides in future studies.

### Atmospheric Implications

For the 20 synthesized AAHPs studied here, we show that their formation kinetics vary only within a factor of 2–3. This implies that an average rate constant could be reasonably applied for this type of bimolecular reaction, which can largely simplify the complexity of predicting AAHPs in kinetic models. However, one should be aware that similar kinetic experiments for AAHP formation in the gas phase would be necessary before extrapolating this result to atmospheric gas-phase AAHP modeling. The hydrolysis experiments show that all of these 20 AAHPs decompose in water but with a hydrolysis rate differing up to 2 orders of magnitude. This implies that the hydrolysis behavior of AAHPs is largely compound-dependent (in contrast to their formation kinetics), and AAHPs may not always show rapid hydrolysis as thought previously.^16^ Such a detailed understanding is critical to accurately determine their major atmospheric removal pathways and environmental lifetime, especially considering that AAHPs can effectively partition into aerosol and cloud droplets or form in the aqueous phase.^36,37^ In addition, the negative correlation between AAHP molecular weight and hydrolysis rate (see Figure S12B) implies that these AAHPs with higher molecular weight are likely to have longer environmental lifetimes, which could be important for health risk assessment studies.

Based on recent developments of high-resolution mass spectrometry techniques, several recent studies reported molecular-level identification of particle-phase organic peroxides.^38,39^ However, these mostly rely on tentative formula assignment as the method of identification, while unambiguous assignments are only possible when authentic standards are available. For the first time with the help of AAHP standards, we unambiguously identified four AAHPs and quantitative estimates are given for two types of monoterpene-SOA generated in a laboratory flowtube reactor. The current study suggests that acetic acid, cis-pinonic acid, and 3-caronic acid are major contributors to monoterpene-AAHPs, but this is likely dependent on specific SOA formation conditions, and further work using additional authentic standards is required to fully characterize the peroxides content at a molecular level in SOA. Although the bimolecular reaction of SCIs with carboxylic acids is considered a representative route leading to dimeric organic peroxides, the importance of this route in SOA chemistry still requires more quantitative understanding, which cannot be revealed based on our one specific condition for monoterpene-SOA generation. Therefore, further ozonolysis experiments that cover a wide range of SOA formation conditions (e.g., aerosol pH and phase state, temperature, and relative humidity) are needed in combination with targeted analysis using synthesized AAHP standards.

The chromatograms of SOA extracts obtained in this study (see Figure 3C–F as examples) illustrate that many isomers with the same exact *m*/*z* can be separated, i.e., elute at different retention times. It is likely that other peroxides and also nonperoxides are among those isomers, and only authentic standards will allow accurate unambiguous structural assignment of these components. Currently, the lack of authentic standards limits the ability of molecular-level identification of organic peroxides in SOA, further preventing quantification and evaluation of their health impact. The methodology presented in this study demonstrates an efficient and versatile method to obtain atmospherically relevant organic peroxide standards. The synthesized organic peroxide standards are also useful to examine ROS activity and toxicity, e.g., in cell culture studies.

## Supporting Information Available

Experimental details, experimental setup and summary of experimental conditions, as well as further results and summary from LC-HRMS chromatograms, MS^2^ spectra, control experiments, hydrolysis experiments, and flowtube experiments (PDF)
