# Supplementary material for: Synthesis and Characterization of Organic Peroxides from Monoterpene-Derived Criegee Intermediates in Secondary Organic Aerosol
Source: Environ Sci Technol. 2024 Feb 7;58(7):3322–31. doi: 10.1021/acs.est.3c07048 (PMC10927166; doi:10.1021/acs.est.3c07048)
Supplement: Supplementary file 1 — es3c07048_si_001.pdf [file es3c07048_si_001.pdf]

Supporting Information

Synthesis and characterization of organic peroxides from monoterpene-derived Criegee intermediates in secondary organic aerosol

Kangwei Li*, Julian Resch, Markus Kalberer*

Department of Environmental Sciences, University of Basel, Basel 4056, Switzerland

Correspondence to: Kangwei Li (kangwei.li@unibas.ch), Markus Kalberer (markus.kalberer@unibas.ch)

This PDF file includes:

Supplementary Text 1 to Text S3

Fig. S1 to S13

Table S1 to S8

References (1-7)

Text S1 Clarification on leftover ozone in our experiments

At ozone concentrations of 500 ppm and a flow of 100 mL/min, if ozone can be fully dissolved in 10 mL acetonitrile (ACN), then the maximum predicted ozone flux in ACN at t = 15 min would be ~3 mM. This is somehow consistent with the plateau observed for these carboxylic acids and AAHPs at 5 min in typical kinetic experiments (Figure 2), which corresponds to ~1 mM ozone flux (at 5 min) vs. 1 mM initial monoterpene, as the stoichiometric proportion has been reported to be 1:1 for the two monoterpenes (α/β-pinene) with ozone in aqueous reaction.[1] The observed plateau after 5 min suggests that monoterpene was fully removed. After that, the dissolved ozone is likely to accumulate until the end of the bubbling process, which would react with other (degradation) compounds and/or would decompose overtime within a few hours.

According to the blank ozone bubbling experiment in pure ACN solution without monoterpene addition (Fig. S2), the steady-state concentration of dissolved ozone in ACN at t = 15 min is measured as ~27 μM, according to the molar absorption coefficient of ozone at 260 nm = 3000 M^{-1} cm^{-1}.[2] This suggests that 99% of gas-phase ozone is actually not dissolved in ACN if no monoterpene is present, as the maximum predicted ozone flux was calculated as ~3 mM at t = 15 min. It is also known that ozone is not stable in aqueous solvent, and the decomposition rate constant of ozone in acetonitrile has been reported as 3.3×10^{-5} s^{-1} at room temperature,[3] suggesting an e-folding lifetime of ozone of 8.4 h, which is an upper limit in our reaction system. The control experiment (Fig. S6) shows the stability of carboxylic acids in the presence of ozone flow, which further suggests the negligible influence of dissolved / leftover ozone on the removal of carboxylic acids.

Text S2 OH radical production during the liquid-phase ozonolysis of monoterpene

OH radicals are generated during the ozonolysis of alkenes via the decay of Criegee intermediates, which may cause further OH-oxidation reactions with carboxylic acids. We assessed such potential unwanted reactions by i) reactivity calculation; ii) OH measurement. Acetonitrile reacts with OH radicals with a rate constant in the liquid phase in the order of 10^6 M^{-1} s^{-1} (see section 4.2.2.1 in https://www.inchem.org/documents/ehc/ehc/ehc154.htm). Therefore, the reactivity of OH with acetonitrile is about 2×10^7 s^{-1} in pure acetonitrile (with a concentration of 19.2 M).

Aqueous phase rate constants of carboxylic acids with OH radicals are in the order of 10^9 M^{-1} s^{-1}.[4] Thus the OH reactivity with the carboxylic acid mixture (total concentration of ~0.2 mM) is calculated as about 2×10^5 s^{-1}, which is about two orders of magnitude lower than that of acetonitrile. This suggests that the majority of OH radicals (ca. 99%) produced during the ozonolysis of monoterpene are scavenged by acetonitrile and that the concentrations of carboxylic acids are not affected by OH.

Additional OH measurements with fluorescence spectroscopy also supported the calculation above. Terephthalic acid (TA) was previously used as fluorescent probe to quantify the OH radical production in solutions, in which TA reacts with OH radical to form a fluorescent product - 2-hydroxyterephthalic acid (TAOH), with a product yield of 0.315 according to Gonzalez et al.[5]

Fluorescence intensities of TAOH standards (0–10 nM) and synthesized AAHPs solutions that contain TA during the ozonolysis of α-pinene and 3-carene were analyzed with a commercial fluorescence spectroscopy (Fluorolog®-3, Horiba Jobin Yvon). Fig. S7 shows typical fluorescence emission spectra of five TAOH standards with concentrations 0–10 nM peaking at ~420 nm (excited at 310 nm). In the two spectra containing the monoterpene/carboxylic acid mixtures, no TAOH fluorescent peak was observed, suggesting a negligible TAOH formation (< 10 nM) from OH-oxidation and therefore negligible OH accumulated concentrations (< 30 nM) during the ozonolysis of monoterpene.

Text S3 Additional details for iodometry-assisted LC-HRMS for the synthesized AAHPs

We obtained synthesized solution containing a mixture of ten different AAHPs for each monoterpene precursor, which were treated following an iodometry-assisted LC-HRMS method. As initially proposed by Zhao et al.,[6] we used this method as a supplementary confirmation that the compounds we synthesized are indeed organic peroxides.

Specifically, one aliquot of synthesized AAHPs (300 μL, in ACN) was mixed with aqueous solution of acetic acid (260 μL, 50 mM in ACN) and camphorsulfonic acid (20 μL, 0.3 mM in ACN, Sigma Aldrich) in an airtight vial, followed by addition of KI solution (20 μL, 600 mM in water, Sigma Aldrich). These samples are named here “KI-treated”. Another aliquot of synthesized AAHPs (300 μL, in ACN) was treated with the same procedures but without the addition of KI solution (replaced with 20 μL water), named hereafter as “non-treated” sample. The addition of acetic acid was to slow down the hydrolysis process, while camphorsulfonic acid was added as an internal standard to make sure that the mass spectrometry ionization efficiency did not change due to the presence of KI. These samples were placed in the dark for 3 hr at room temperature before being analyzed by LC-HRMS, and the results are shown in Fig. S3.

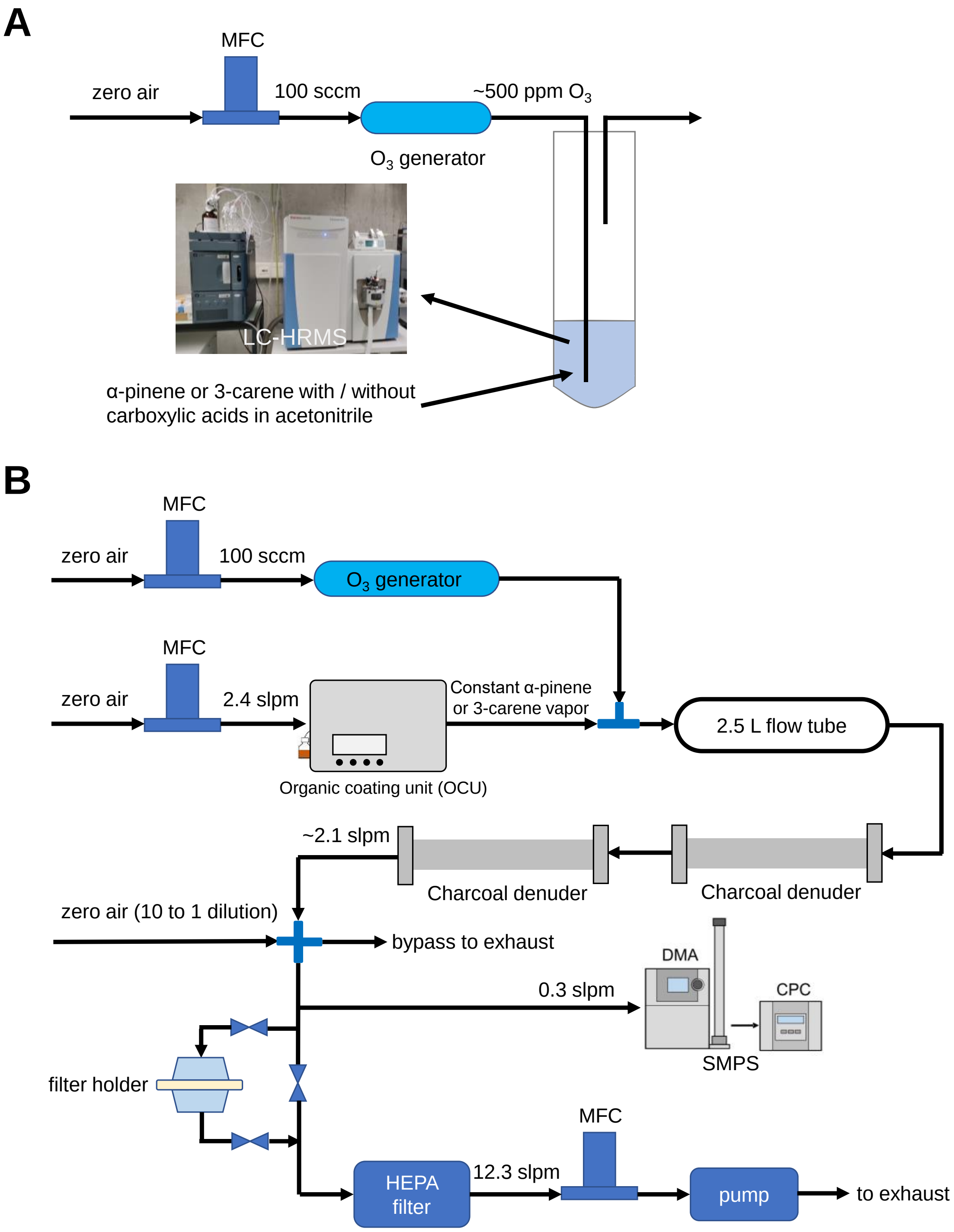

Fig. S1 (A) Experimental setup of the ozone bubbling experiments; (B) flowtube setup to generate and collect α-pinene SOA and 3-carene SOA.

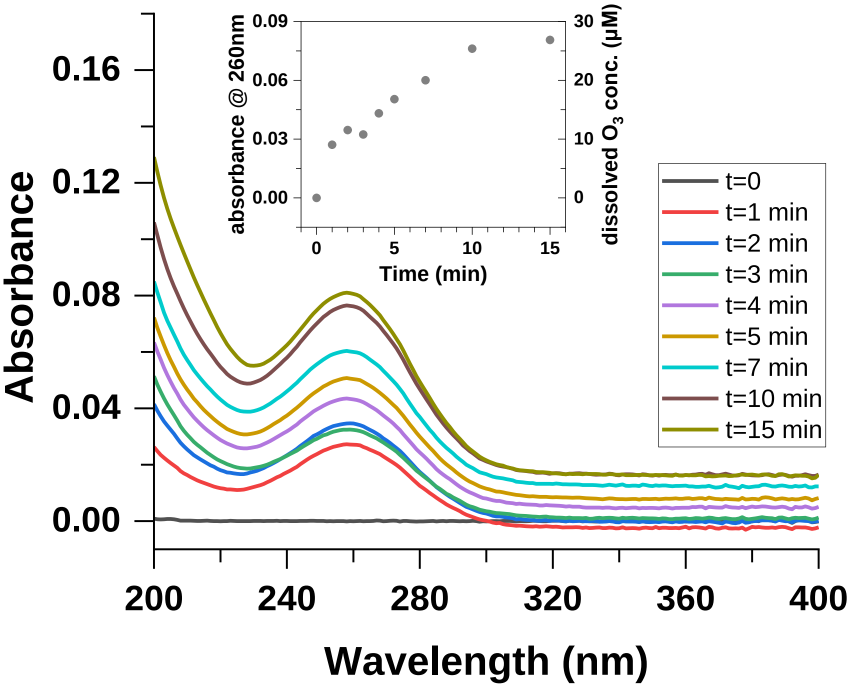

Fig. S2 UV-Vis spectra of ozone during a control experiment without any organic compounds (expt 20 in Table S2) while bubbling ozone through acetonitrile.

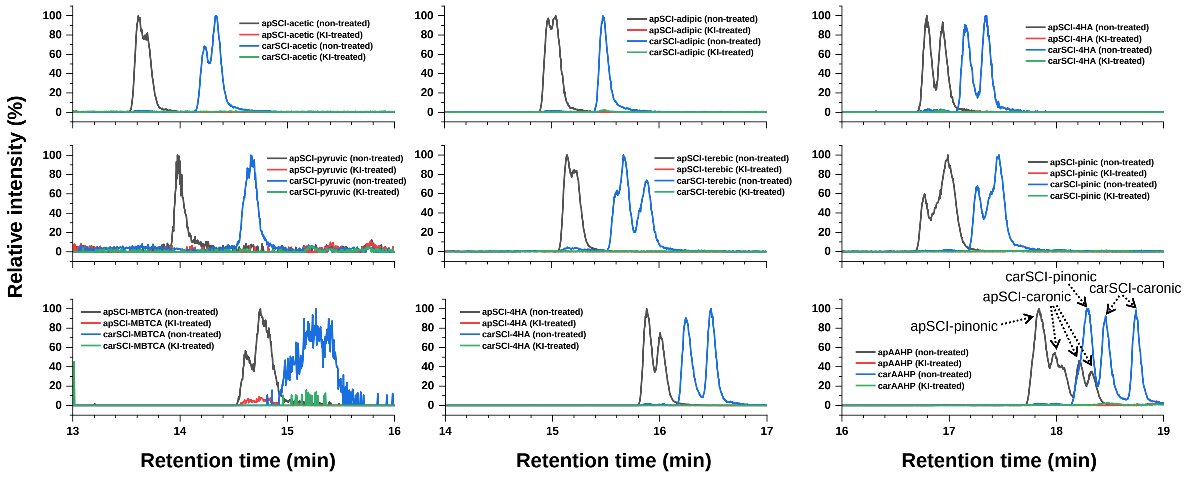

Fig. S3 Extracted ion chromatograms (EIC) for synthesized AAHPs solutions that were treated by KI and non-treated. Their retention time (RT) and other details are summarized in Table S5.

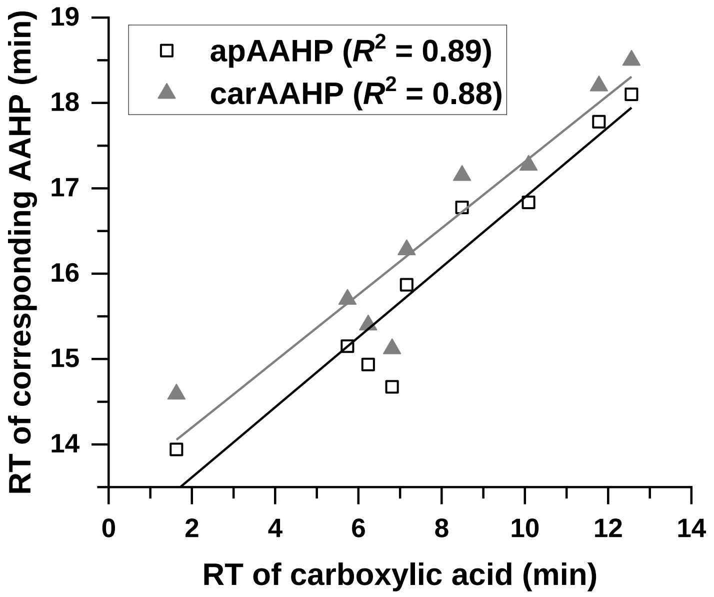

Fig. S4 Correlation of retention times (RT) of carboxylic acids and corresponding AAHPs. The average of RT was used when two or more peaks were detected in the EIC.

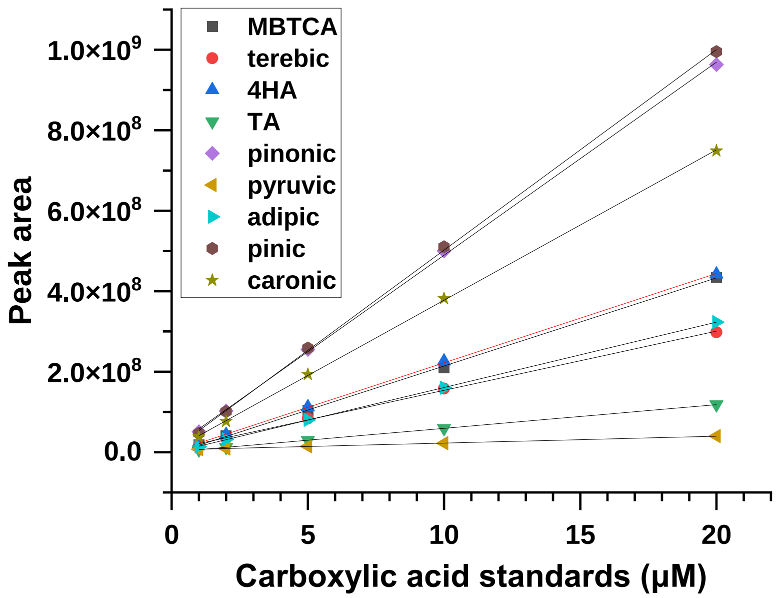

Fig. S5 Five-point calibration curve for all carboxylic acids (1, 2, 5, 10 and 20 µM).

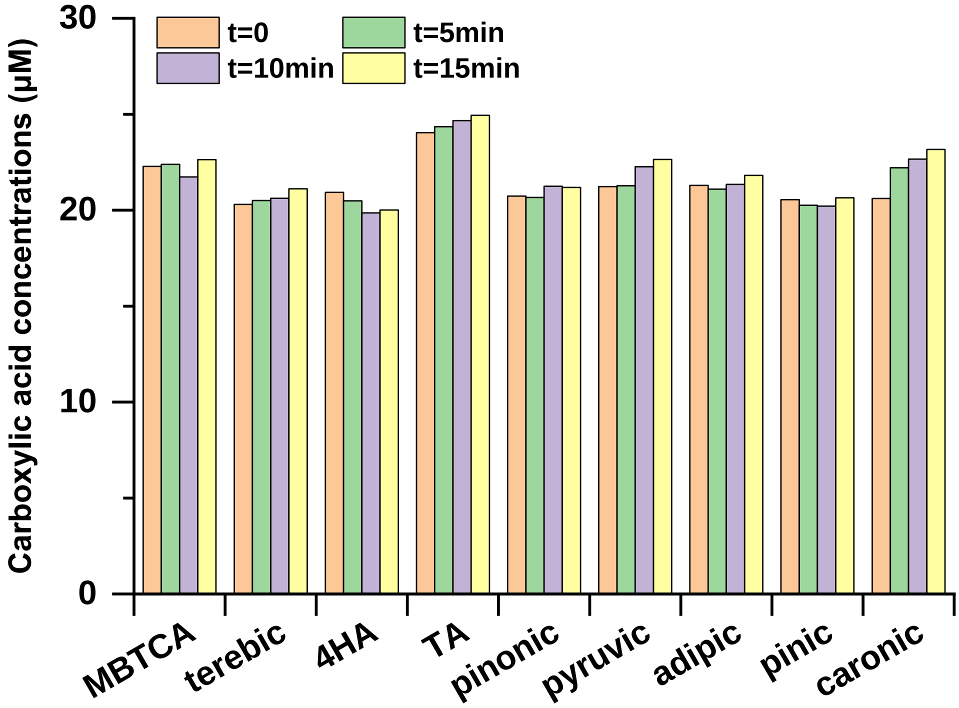

Fig. S6 The concentration of nine carboxylic acids over time (0–15 min). This control experiment (expt 19 in Table S2) shows that the carboxylic acids in ACN (without monoterpene) are stable in the presence of ozone bubbling through the impinger.

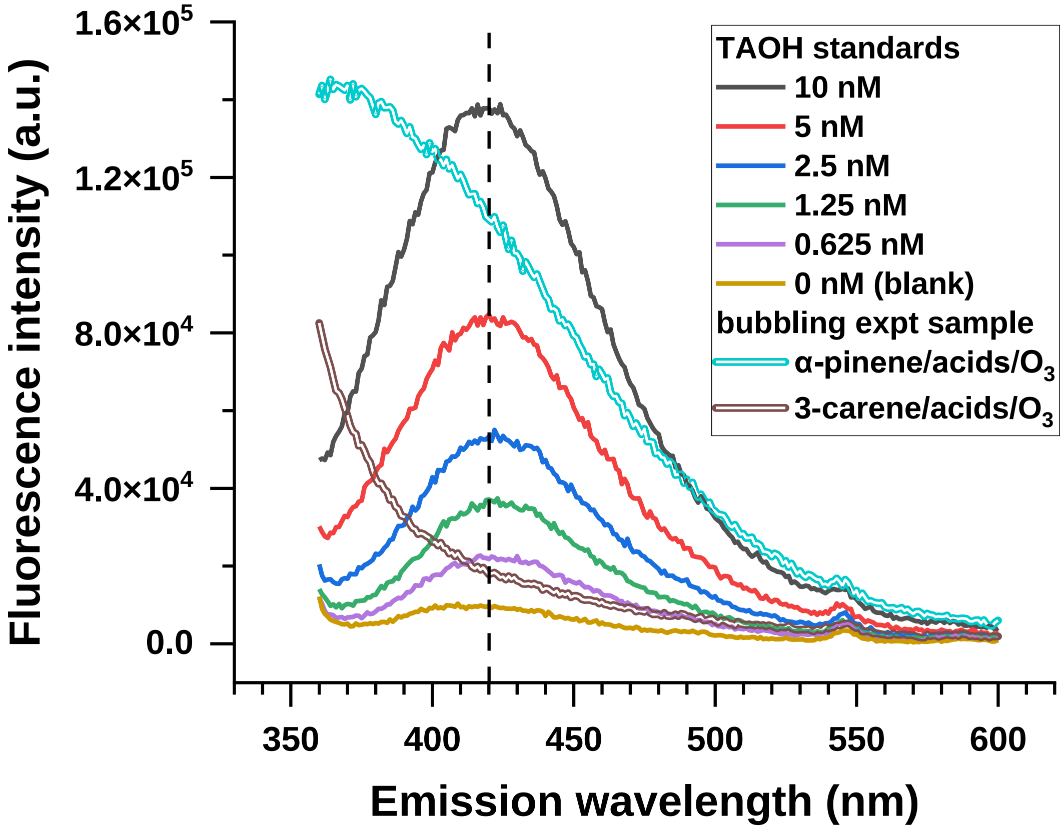

Fig. S7 Fluorescence emission spectra (excited at 310 nm) for TAOH standards (0−10 nM) as well as two samples with O_3, acids and monoterpenes from bubbling expt 15 and 16 (see Table S2) that contain TA during the ozonolysis of monoterpene. No detectable TAOH was formed in the AAHPs samples.

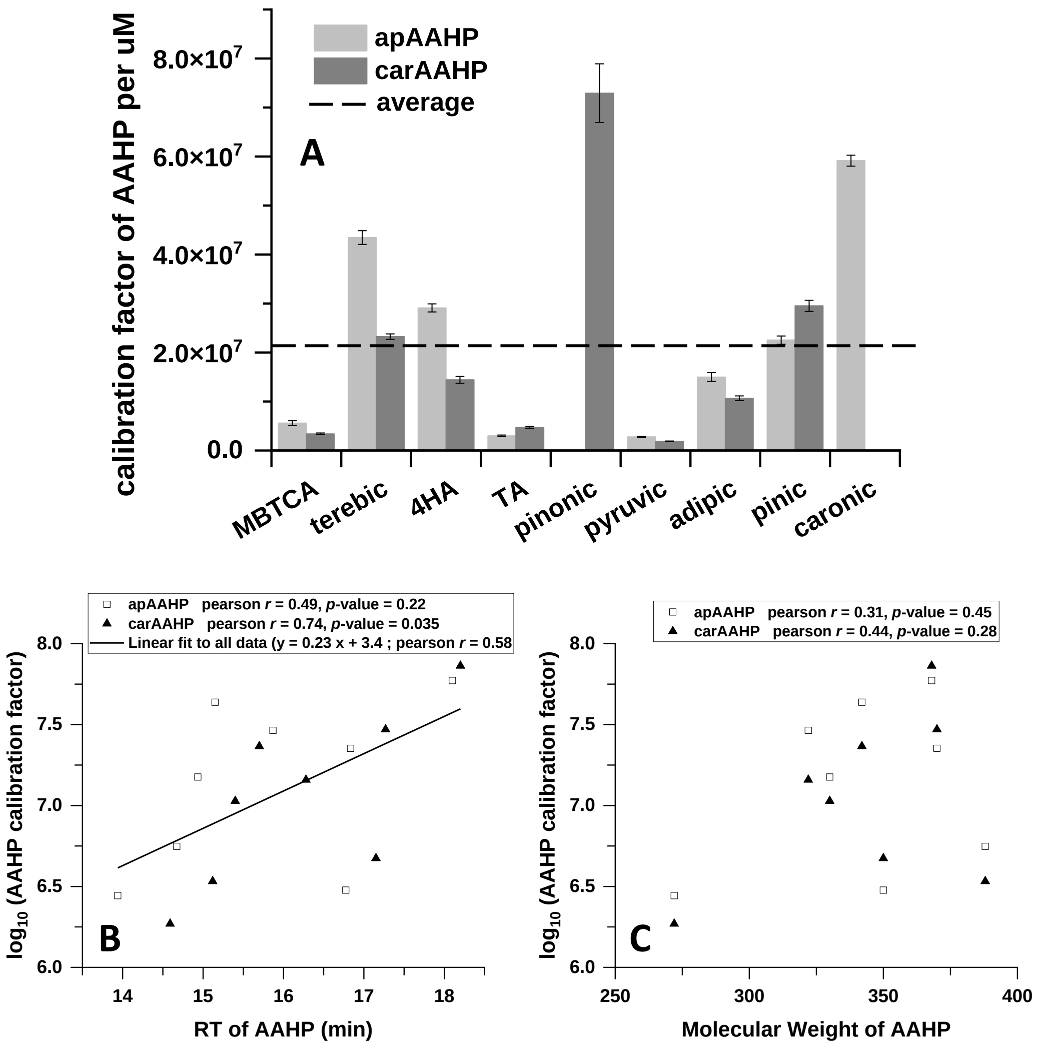

Fig. S8 (A) Calibration factors for 16 AAHPs that are equal to the slope of the linear fits in Fig. 2F and Fig. 2L. Correlation of 16 AAHP calibration factors with (B) retention time (RT) of the AAHP; and with (C) molecular weight of the AAHP. The linear fit in (B) is used to estimate some AAHP calibration factors for quantification as shown in Table S8

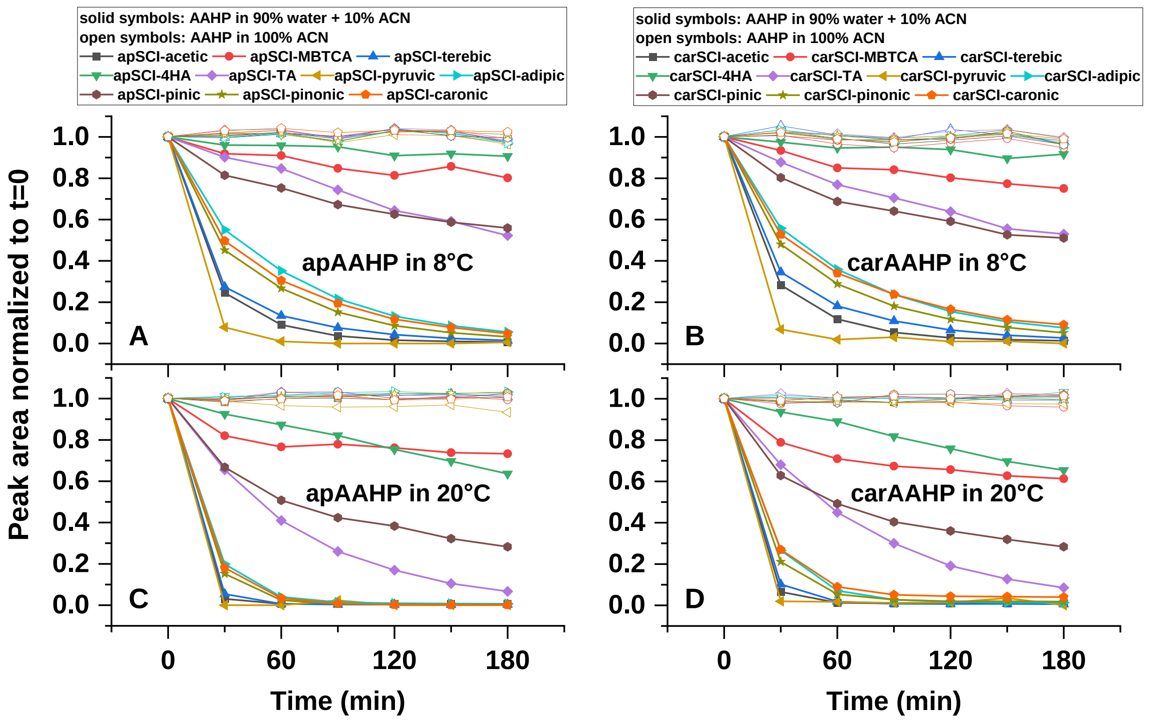

Fig. S9 Temporal variations of (A, C) apAAHP and (B, D) carAAHP in two solvents (90% water + 10% ACN; 100% ACN) over three hours at 8 °C and 20 °C, respectively.

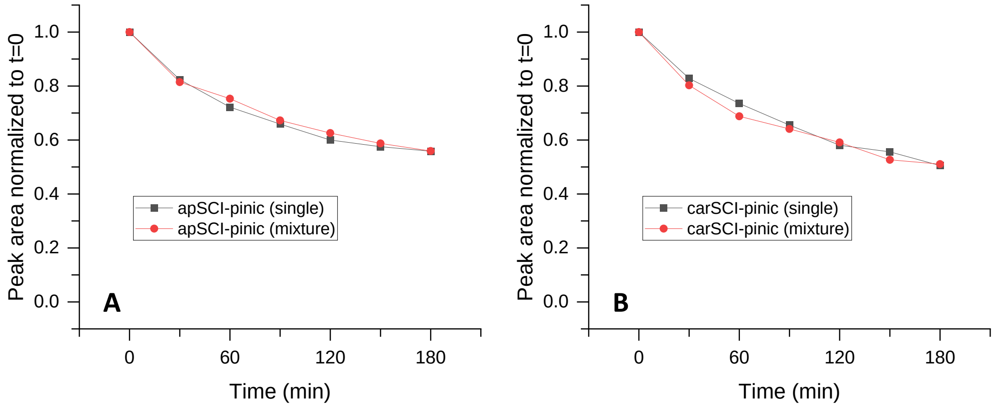

Fig. S10 Comparison of AAHP hydrolysis (90% water + 10% ACN) from synthesized solutions containing one single AAHP vs. a mixture of AAHPs. (A) apSCI-pinic; (B) carSCI-pinic

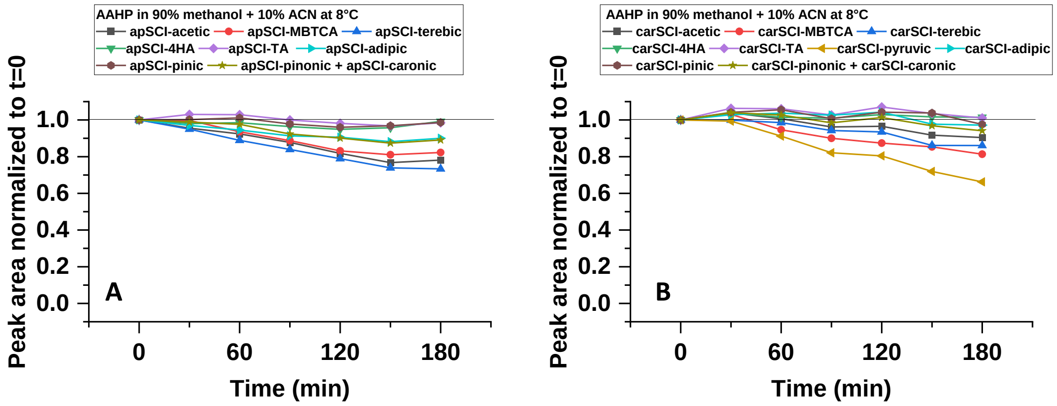

Fig. S11 Stability of AAHPs in methanol-dominant solvent (90% methanol + 10% ACN)

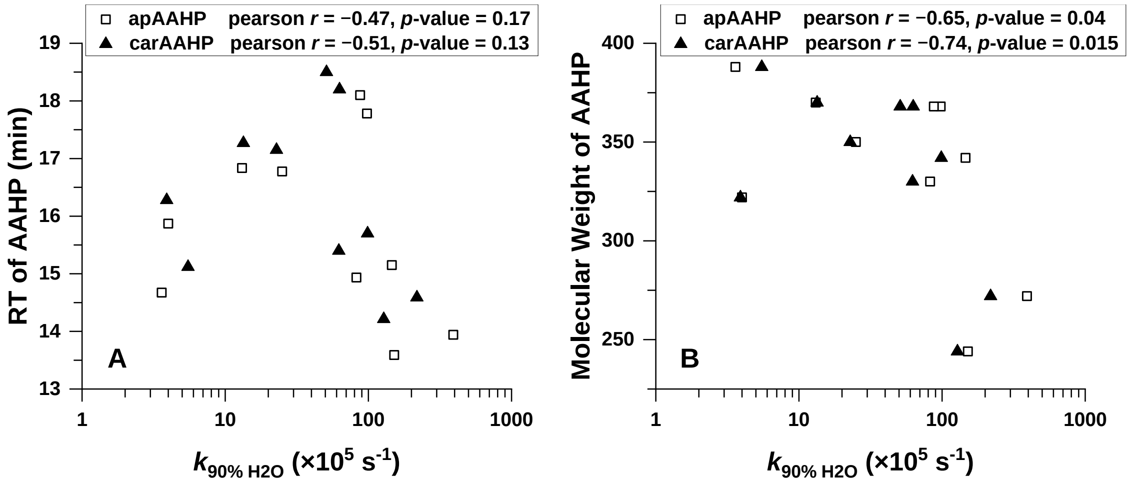

Fig. S12 Correlation of AAHP hydrolysis rate (measured at 20 °C) with (A) retention time (RT) of AAHP; and with (B) molecular weight of AAHP.

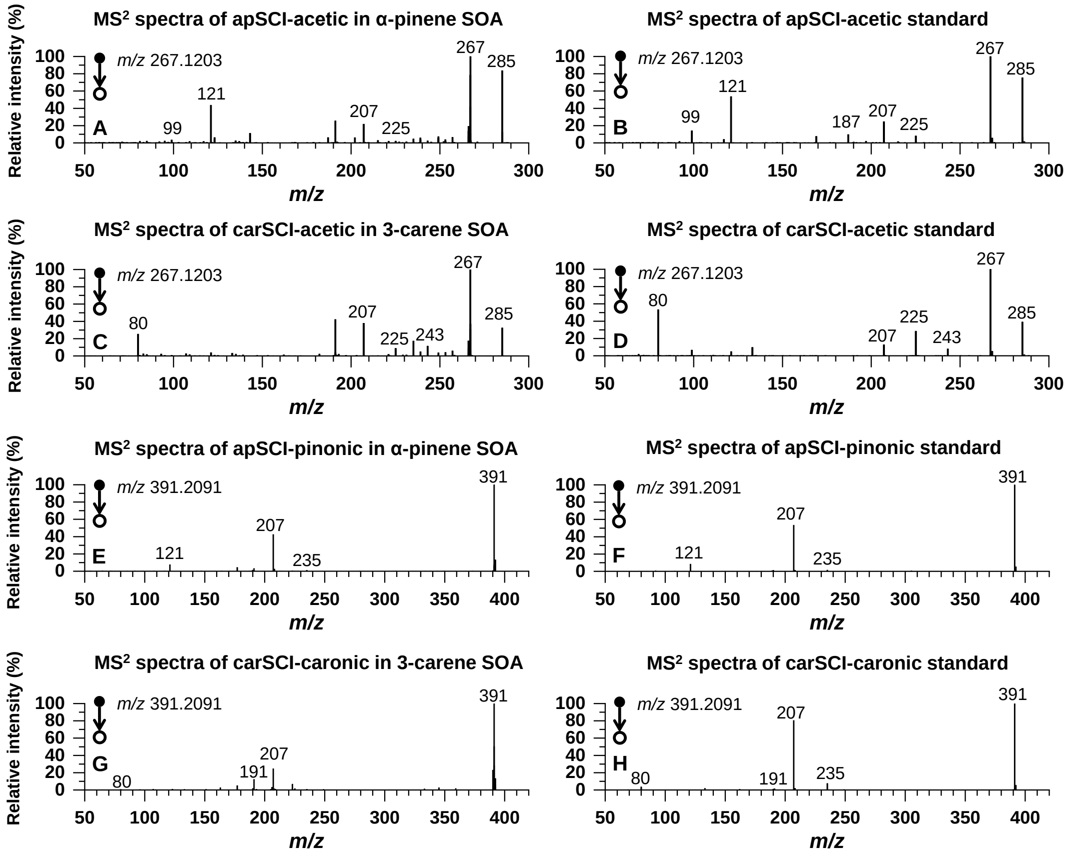

Fig. S13 MS^2 spectra of four AAHPs identified in laboratory-generated SOA (left column) as shown in Fig. 3C–3F, and compared with AAHPs standards at identical retention times (right column) as shown in Fig. 3A–3B. (A, B) apSCI-acetic; (C, D) carSCI-acetic; (E, F) apSCI-pinonic; (G, H) carSCI-caronic. These MS^2 spectra are obtained under higher-energy collisional dissociation (HCD) of 10.

Table S1 LC-HRMS measurement uncertainties obtained from daily injection of a HPLC Gradient System Diagnostics Mix (Sigma Aldrich) containing five compounds, including methyl 4-hydroxybenzoate ($C_8H_8O_3$), ethyl 4-hydroxybenzoate ($C_9H_{10}O_3$), propyl 4-hydroxybenzoate ($C_{10}H_{12}O_3$), butyl 4-hydroxybenzoate ($C_{11}H_{14}O_3$) and heptyl paraben ($C_{14}H_{20}O_3$)

Formula		$C_8H_8O_3$	$C_9H_{10}O_3$	$C_{10}H_{12}O_3$	$C_{11}H_{14}O_3$	$C_{14}H_{20}O_3$
RT (min)		11.85	14.2	16.3	18.07	21.91
Uncertainty (1σ)	neg. mode	2.5%	3.2%	3.7%	3.3%	2.3%
	pos. mode	3.5%	1.9%	1.0%	1.5%	2.7%

Table S2 Summary of experimental conditions for all ozone bubbling experiments. The ozone bubbling flow rate is 100 mL min^{-1} with an ozone concentration of ~500 ppm.

expt ID	initial BVOC	initial carboxylic acid	initial vol (mL)	total expt time (min)	expt purpose
1	10mM α-pinene	1mM cis-pinonic acid	5	5	AAHP synthesis
2	10mM 3-carene	1mM cis-pinonic acid	5	5	
3	10mM α-pinene	/	5	5	control expt
4	10mM 3-carene	/	5	5	
5	1mM α-pinene	0.1mM 3-caronic acid	10	20	AAHP synthesis
6	1mM 3-carene	0.1mM 3-caronic acid	10	20	
7	1mM α-pinene	0.1mM terebic acid	10	20	
8	1mM 3-carene	0.1mM terebic acid	10	20	
9	1mM α-pinene	0.1mM cis-pinic acid	10	20	
10	1mM 3-carene	0.1mM cis-pinic acid	10	20	
11	1mM α-pinene	0.1mM MBTCA	10	20	
12	1mM 3-carene	0.1mM MBTCA	10	20	
13	1mM α-pinene	/	10	20	control expt
14	1mM 3-carene	/	10	20	
15	1mM α-pinene	10 acids (each ~0.02mM)	10	15	kinetic of carboxylic acid with SCI, AAHP synthesis and hydrolysis
16	1mM 3-carene	10 acids (each ~0.02mM)	10	15	
17	1mM α-pinene	/	10	15	control expt
18	1mM 3-carene	/	10	15	
19	/	10 acids (each ~0.02mM)	10	15	
20	/	/	10	15	

[a] The ten carboxylic acids include acetic acid (acetic), pyruvic acid (pyruvic), terebic acid (terebic), adipic acid (adipic), 3-methyl-1,2,3-butanetricarboxylic acid (MBTCA), 4-hydroxybenzoic acid (4HA), terephthalic acid (TA), cis-pinic acid (pinic), cis-pinonic acid (pinonic) and 3-caronic acid (caronic).

[b] For expt 15, the initial concentration of TA was quantified as 45 μM, while all the other carboxylic acids were quantified within 20–22 μM. For expt 16, the initial concentrations of all carboxylic acids were quantified within 20–22 μM.

Table S3 Comparison of monoterpene-SOA samples that were extracted in two different ways, with a solvent volume difference of 15. (a) Extraction with drying and reconstitution in 100 μL ACN; (b) extraction in 1.5 mL ACN without drying

	Name	Formula	(a) drying and reconstitution	(b) non-drying	
			peak area	peak area	ratio
α-pinene SOA	terebic	$C_7H_{10}O_4$	1.40E+07	1.26E+06	11.1
	pinic	$C_9H_{14}O_4$	1.01E+09	7.59E+07	13.3
	pinonic	$C_{10}H_{16}O_3$	8.39E+08	7.26E+07	11.6
	10-hydroxypinonic	$C_{10}H_{16}O_4$	3.65E+08	2.90E+07	12.6
	MBTCA	$C_8H_{12}O_6$	3.51E+06	2.76E+05	12.7
3-carene SOA	terebic	$C_7H_{10}O_4$	1.56E+07	1.46E+06	10.7
	caric	$C_9H_{14}O_4$	1.45E+09	1.25E+08	11.6
	caronic	$C_{10}H_{16}O_3$	1.67E+09	1.64E+08	10.2
	10-hydroxy-3caronic	$C_{10}H_{16}O_4$	7.64E+08	6.86E+07	11.1

Table S4 Summary of structure, formula, *m/z* and retention time (RT) for all carboxylic acids from Fig. 1.

Name	structure	formula [M]	*m/z* [M-H]$^-$	RT (min)
acetic acid (acetic)	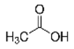	$C_2H_4O_2$	59.0139	N.D.
pyruvic acid (pyruvic)	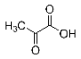	$C_3H_4O_3$	87.0088	1.63
terebic acid (terebic)	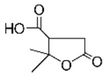	$C_7H_{10}O_4$	157.0506	5.74
adipic acid (adipic)	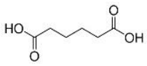	$C_6H_{10}O_4$	145.0506	6.12, 6.35
3-methyl-1,2,3-butanetricarboxylic acid (MBTCA)	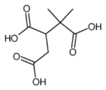	$C_8H_{12}O_6$	203.0561	6.81
4-hydroxybenzoic acid (4HA)	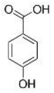	$C_7H_6O_3$	137.0244	7.16
terephthalic acid (TA)	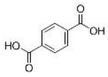	$C_8H_6O_4$	165.0193	8.49
cis-pinic acid (pinic)	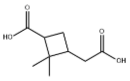	$C_9H_{14}O_4$	185.0819	10.09
cis-pinonic acid (pinonic)	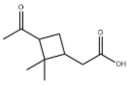	$C_{10}H_{16}O_3$	183.1027	11.78
3-caronic acid (caronic)	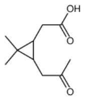	$C_{10}H_{16}O_3$	183.1027	12.56

Table S5 Summary of formula, *m/z* and RT for all AAHPs from Fig. 1.

Name of AAHPs	formula [M]	*m/z* $[M+Na]^+$	apAAHP RT (min)	carAAHP RT (min)	other detected adducts
apSCI-acetic, carSCI-acetic	$C_{12}H_{20}O_5$	267.1203	13.56, 13.62	14.16, 14.27	$[M+NH_4]^+$, $[M+K]^+$
apSCI-pyruvic, carSCI-pyruvic	$C_{13}H_{20}O_6$	295.1152	13.94	14.59	$[M+NH_4]^+$, $[M+K]^+$
apSCI-terebic, carSCI-terebic	$C_{17}H_{26}O_7$	365.1571	15.1, 15.2	15.59, 15.81	$[M+NH_4]^+$, $[M+K]^+$
apSCI-adipic, carSCI-adipic	$C_{16}H_{26}O_7$	353.1571	14.9, 14.97	15.4	$[M+NH_4]^+$, $[M+K]^+$, $[M-H]^-$
apSCI-MBTCA, carSCI-MBTCA	$C_{18}H_{28}O_9$	411.1626	14.52, 14.67, 14.83	14.88, 15.15, 15.33	$[M+NH_4]^+$, $[M+K]^+$, $[M-H]^-$
apSCI-4HA, carSCI-4HA	$C_{17}H_{22}O_6$	345.1309	15.81, 15.93	16.17, 16.39	$[M+NH_4]^+$, $[M+K]^+$, $[M-H]^-$
apSCI-TA, carSCI-TA	$C_{18}H_{22}O_7$	373.1258	16.7, 16.85	17.05, 17.25	$[M+NH_4]^+$, $[M+K]^+$, $[M-H]^-$
apSCI-pinic, carSCI-pinic	$C_{19}H_{30}O_7$	393.1884	16.72, 16.95	17.17, 17.37	$[M+NH_4]^+$, $[M+K]^+$, $[M-H]^-$
apSCI-pinonic, carSCI-pinonic	$C_{20}H_{32}O_6$	391.2091	17.78	18.2	$[M+NH_4]^+$, $[M+K]^+$
apSCI-caronic, carSCI-caronic	$C_{20}H_{32}O_6$	391.2091	17.93, 18.01, 18.16, 18.3	18.36, 18.64	$[M+NH_4]^+$, $[M+K]^+$

Table S6 Relative rate constant for the two C10 SCI (apSCI or carSCI) with different carboxylic acids. k_{obs} of each acid is also normalized to k_{obs} of MBTCA.

		MBTCA	Terebic	4HA	TA	Pinonic	Pyruvic	Adipic	Pinic	Caronic
apSCI	k_{obs} ($\times10^{-4}$ s^{-1})	37.1±0.9	19.1±0.5	22.3±0.6	36.4±0.8	/	37.9±1.1	37.5±0.9	31.9±1.5	17.3±0.5
	Normalized k_{obs}	1.00±0.02	0.51±0.01	0.60±0.02	0.98±0.02	/	1.02±0.03	1.01±0.03	0.86±0.04	0.47±0.01
carSCI	k_{obs} ($\times10^{-4}$ s^{-1})	22.0±0.7	9.9±0.3	12.9±0.6	18.1±0.6	7.1±0.6	74.8±5.3	28.9±0.6	16.5±0.5	/
	Normalized k_{obs}	1.00±0.03	0.45±0.01	0.59±0.03	0.82±0.03	0.32±0.03	3.40±0.24	1.32±0.03	0.75±0.02	/

Table S7 Comparison of hydrolysis rates and e-folding lifetimes for two individual AAHPs obtained from two studies.

		apSCI-pinonic		apSCI-adipic	
		k ($\times10^{-5}$ s^{-1})	lifetime (min)	k ($\times10^{-5}$ s^{-1})	lifetime (min)
Zhao et al.[7]	20 °C	99.1 ± 21.4	17 ± 4	72.4 ± 9.9	23 ± 3
	8 °C	34.2 ± 7.4	49 ± 11	23.6 ± 3.2	71 ± 10
This study	20 °C	97.8 ± 2.4	17 ± 0.4	82.5 ± 3.1	20 ± 1
	8 °C	33.2 ± 0.7	50 ± 1	27.5 ± 0.4	61 ± 1

Note: The hydrolysis rates in Zhao et al.[7] have been adjusted to the conditions (temperature of 20 °C or 8 °C, pH=4.96, water content) used in this study according to their temperature- and pH-dependence. Since their hydrolysis rates were obtained by a dilution in 98% water content, these were normalized by a factor of 0.9/0.98 to match the 90% water content used in this study.

Table S8 Initial conditions for the generation of two monoterpene-SOA in a flowtube reactor, with their AAHPs concentrations estimated.

type of SOA	initial BVOC (ppm)	initial O_3 (ppm)	measured SOA conc. (μg m^{-3})	collected SOA mass per filter (μg)	semi-quantified AAHP per filter (ng)		two identified AAHPs mass fraction to SOA (%)
α-pinene SOA					apSCI-acetic	apSCI-pinonic	
	61.3	20	~800	29.5	2.9	91.6	0.32
3-carene SOA					carSCI-acetic	carSCI-caronic	
	44	20	~1600	59	8.2	177.4	0.31

Note: The initial BVOC concentration is roughly estimated based on their vapor pressure at 25 °C, and the initial O_3 concentration is measured by an ozone analyzer (Thermo 49i). The SOA mass concentration is measured by SMPS (TSI 3936) with an assumption of SOA density of 1.2 g cm^{-3}, while the collected SOA mass per filter is calculated by sampling flow rate of 12.3 LPM with a sampling duration of 180 s.

References

(1) Zhang, X.; Chen, Z.; Wang, H.; He, S.; Huang, D. An important pathway for ozonolysis of alpha-pinene and beta-pinene in aqueous phase and its atmospheric implications. *Atmospheric Environment* **2009**, *43* (29), 4465-4471. DOI: 10.1016/j.atmosenv.2009.06.028.

(2) Levanov, A. V.; Isaikina, O. Y.; Tyutyunnik, A. N.; Antipenko, E. E.; Lunin, V. V. Molar absorption coefficient of ozone in aqueous solutions. *Journal of Analytical Chemistry* **2016**, *71* (6), 549-553. DOI: 10.1134/s1061934816060083.

(3) Panich, N. M.; Ershov, B. G. Solubility and stability of ozone in acetonitrile. *Journal of Molecular Liquids* **2021**, *340*. DOI: 10.1016/j.molliq.2021.117318.

(4) Herrmann, H.; Schaefer, T.; Tilgner, A.; Styler, S. A.; Weller, C.; Teich, M.; Otto, T. Tropospheric aqueous-phase chemistry: kinetics, mechanisms, and its coupling to a changing gas phase. *Chem Rev* **2015**, *115* (10), 4259-4334. DOI: 10.1021/cr500447k.

(5) Gonzalez, D. H.; Kuang, X. M.; Scott, J. A.; Rocha, G. O.; Paulson, S. E. Terephthalate Probe for Hydroxyl Radicals: Yield of 2-Hydroxyterephthalic Acid and Transition Metal Interference. *Analytical Letters* **2018**, *51* (15), 2488-2497. DOI: 10.1080/00032719.2018.1431246.

(6) Zhao, R.; Kenseth, C. M.; Huang, Y.; Dalleska, N. F.; Seinfeld, J. H. Iodometry-Assisted Liquid Chromatography Electrospray Ionization Mass Spectrometry for Analysis of Organic Peroxides: An Application to Atmospheric Secondary Organic Aerosol. *Environ Sci Technol* **2018**, *52* (4), 2108-2117. DOI: 10.1021/acs.est.7b04863.

(7) Zhao, R.; Kenseth, C. M.; Huang, Y.; Dalleska, N. F.; Kuang, X. M.; Chen, J.; Paulson, S. E.; Seinfeld, J. H. Rapid Aqueous-Phase Hydrolysis of Ester Hydroperoxides Arising from Criegee Intermediates and Organic Acids. *J Phys Chem A* **2018**, *122* (23), 5190-5201. DOI: 10.1021/acs.jpca.8b02195.
